# The Association Between Dietary Iron, the SNP of the JAZF1 rs864745, and Glucose Metabolism in a Chinese Population

**DOI:** 10.3390/nu16223831

**Published:** 2024-11-08

**Authors:** Zihan Hu, Hongwei Liu, Baozhang Luo, Chunfeng Wu, Changyi Guo, Zhengyuan Wang, Jiajie Zang, Fan Wu, Zhenni Zhu

**Affiliations:** 1 School of Public Health, Fudan University, Shanghai 200032, China; zhhu24@m.fudan.edu.cn (Z.H.); hwliu23@m.fudan.edu.cn (H.L.); 2 Division of Health Risk Factors Monitoring and Control, Shanghai Municipal Center for Disease Control and Prevention, Shanghai 200336, China; luobaozhang@scdc.sh.cn (B.L.); wucunfeng@scdc.sh.cn (C.W.); guochangyi@scdc.sh.cn (C.G.); wangzhengyuan@scdc.sh.cn (Z.W.); zangjiajie@scdc.sh.cn (J.Z.)

**Keywords:** dietary iron, rs864745, single nucleotide polymorphism, sex differences, elevated fasting glucose, glucose metabolism

## Abstract

Objectives: Dysglycemia is prevalent in China; previous studies had shown that dietary iron was associated with glucose metabolism, and rs864745 was also related to it. The objective of this study is to investigate the association between dietary iron, the SNP of the *JAZF1* rs864745, and glucose metabolism among Chinese adults. Methods: 3298 participants (1584 males and 1714 females) were recruited and underwent physical measurements, laboratory tests, and genotyping. All surveys were conducted by qualified public health professionals. Dietary iron was assessed using the 3-day 24 h dietary recall method and condiment weight records. Genotyping for rs864745 was performed using the SNaPshot Multiplex System. Results: After adjusting for covariates, a significant trend was found between the dietary iron and elevated fasting glucose (*p* = 0.012), whereas no such trend was observed for the rs864745 (*p* = 0.932). Among the male participants, the risk of elevated fasting glucose was associated with both dietary iron (compared to the lowest quartile, the ORs with 95% CIs for elevated fasting glucose in Q2,Q3, and Q4 were 1.52 (1.01, 2.45), 1.73 (1.05, 3.00), and 2.49 (1.33, 4.74), respectively) and the rs864745 (OR = 2.15 (1.02, 4.51)), and an interaction effect between them was observed (*p* = 0.041), which was absent in females (*p* = 0.999 and *p* = 0.131, respectively). Stratified by the SNP rs864745, the males without the C allele had a linear risk increase with iron (*p* = 0.018), while the C allele carriers did not. Additionally, ferritin and the rs864745 were associated with the AST-to-ALT ratio (*p* = 0.005 and *p* = 0.048, respectively). Conclusions: Our study found that dietary iron and the SNP rs864745 interacted and were associated with elevated fasting glucose in Chinese males and absent in females. In addition, the presence of a C allele on rs864745 showed higher risks of elevated fasting glucose regardless of the consumption of dietary iron among the males.

## 1. Introduction

Dysglycemia poses a significant public health challenge. According to estimates from the International Diabetes Federation (IDF), the prevalence of diabetes among adults aged 20–79 years globally stood at 10.5% (537 million cases) in 2021 [1,2]. Notably, China ranks first globally in both the number of diagnosed diabetics (140 million) and those undiagnosed (72.83 million), with an alarming undiagnosed rate of 51.7%. Data from the China Chronic Disease and Risk Factors Surveillance (CCDRFS), employing the diagnostic criteria set by the American Diabetes Association (ADA), reveals that the prevalence of diabetes among adults in mainland China is 12.4%, and the prevalence of prediabetes was 38.1% [3], underscoring the gravity of the situation.

The occurrence of dysglycemia is a consequence of the combined effects of genetics and the environment [4], with iron playing a direct role in this abnormality [5]. Metabolomic studies show that high dietary iron and increased body iron stores activate inflammatory responses, contributing to insulin resistance (IR) [6]. Epidemiological analyses and Mendelian randomization studies have consistently demonstrated an association between these factors and the risk of elevated fasting blood and developing type 2 diabetes (T2D) [7]. Furthermore, there are notable sex and genotype disparities in the risk of dysglycemia arising from increased iron. Studies have consistently revealed that, compared to females, males are more prone to an elevated risk of T2D in response to increased dietary iron [8,9]. Moreover, individuals possessing distinct genotypes exhibit varied sensitivities to variations in dietary iron [10].

*JAZF Zinc Finger 1 (JAZF1)*, a multifunctional regulatory factor located on human chromosome 7p15.2 [11], serves as a pivotal modulator of endoplasmic reticulum (ER) stress and ribosome biogenesis [12,13]. Research has elucidated that, as a metabolic regulator, *JAZF1* exerts its influence through diverse mechanisms, modulating lipid synthesis and lipolysis, thereby participating in the intricate regulation of glucose/lipid metabolism and inflammatory responses [14,15,16]. There are numerous well-established single nucleotide polymorphisms (SNPs) of *JAZF1* that have been implicated in diseases related to glucose metabolism, with rs864745 being of particular interest [17]. Specifically, rs864745, located at chr7:28140937 (GRCh38.p14), represents an intron variant within the *JAZF1* gene and has two allelic variants: T and C. Genome-wide association studies (GWAS) have demonstrated that variations at the rs864745 locus are correlated with T2D across various ethnicities, including Europeans, Americans, and Asians. Notably, disparities in the risk allele and the minor allele frequency (MAF) are evident among different human races [18,19].

Despite extensive research on IR, glucose metabolism, and the role of genes in these conditions, the proximate causes, genetic susceptibility mechanisms, and interactions between dietary iron and genetic variations remain underexplored. Dietary changes and the rs864745-C polymorphism significantly alter *JAZF1* mRNA expression in the liver, impacting hepatic function and metabolism [18,20]. Additionally, iron excess is a known factor for liver injury [21]. The precise contribution of the combination of the rs864745 allele and iron to the risk of glucose metabolism remains uncertain, with a lack of evidence from the Chinese population. The objective of this study is to investigate the association between dietary iron and glucose metabolism, as well as its interaction with the rs864745 locus in the *JAZF1* gene.

## 2. Materials and Methods

### 2.1. Study Population

**Figure 1:**
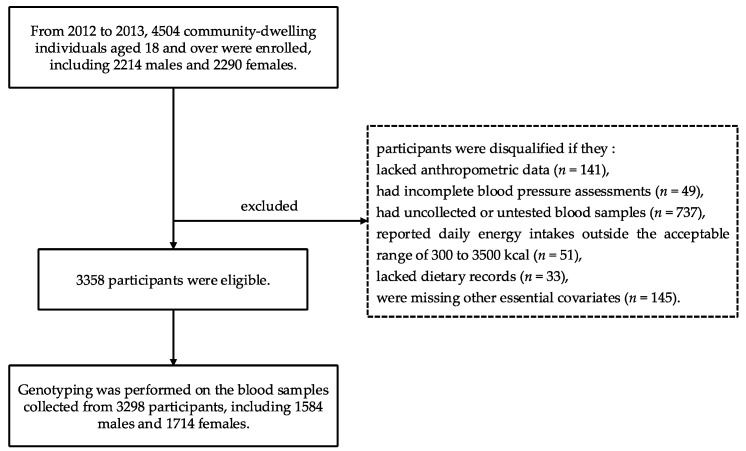
Flow chart of the study participants.

On the basis of the Shanghai Diet and Health Survey (SDHS), between 2012 and 2013, a comprehensive random sampling approach was employed to select 4504 community-dwelling individuals aged 18 and above (consisting of 2214 males and 2290 females) from various communities across Shanghai, China. Subsequently, a household survey and blood sample collection were conducted for these participants. Participants were disqualified if they lacked anthropometric data (*n =* 141), had incomplete blood pressure assessments (*n* = 49), had uncollected or untested blood samples (*n* = 737), reported daily energy outside the acceptable range of 300 to 3500 kcal (*n* = 51), lacked dietary records (*n* = 33), or were missing other essential covariates (*n =* 145). As a result, a final cohort of 3358 eligible participants was established for this study. Notably, genotyping for the SNP rs864745 within the *JAZF1* gene was performed in 3298 of these participants (1584 males and 1714 females) (Figure 1).

### 2.2. Dietary Assessment

Food consumption data, encompassing a comprehensive range of dietary items consumed both domestically and externally, including staples, side dishes, snacks, fruits, alcoholic beverages, non-alcoholic drinks, and nutritional supplements, were meticulously gathered through the 3-day 24 h dietary recall method (spanning 1 weekend day and 2 weekdays), which was meticulously employed to offer a more accurate assessment of iron compared to food frequency questionnaire (FFQ). This rigorous data collection process was conducted by qualified public health professionals from local community healthcare centers, ensuring the validity and comprehensiveness of the dietary information. Additionally, at the beginning and end of the 3-day survey period, the weights of various primary condiments, including edible oils, salt, and monosodium glutamate (MSG), were recorded and quantified. The daily average dietary of energy and iron for each individual was precisely calculated, with the Chinese Food Composition Table serving as the authoritative reference [22]. It is crucial to emphasize that the iron content derived from dietary supplements was not factored into the total dietary iron calculation, given the minimal utilization of iron supplements among the Chinese population [23].

### 2.3. Laboratory Measurements

Venous blood samples were obtained from participants by trained investigators at local community healthcare centers after an overnight 12 h fast, utilizing standardized instruments. These samples were immediately processed for various biochemical assessments. Specifically, the HITACHI 7080 Automatic Biochemical Analyzer, equipped with reagents from Wako Pure Chemical Industries, Ltd. (Tokyo, Japan), was employed to determine fasting plasma glucose (FPG), Glycated Hemoglobin A1c (HbA1c), aspartate aminotransferase (AST), and alanine aminotransferase (ALT) levels. Additionally, a Chemiluminescence Immune Detection System (ACCESS 2, Los Angeles, Beckman Coulter, CA, USA) was utilized to measure ferritin concentrations and fasting insulin concentrations. All these analyses were conducted in the laboratory of the Shanghai Municipal Center for Disease Control and Prevention during the period from 2012 to 2013.

### 2.4. Genotyping

During the fieldwork conducted between 2012 and 2013, white blood cells were promptly stored at a temperature of −80 °C upon collection to preserve their integrity. In 2018, the DNA was extracted from these cells utilizing the magnetic bead method incorporated in the Universal Genomic DNA Extraction Kit (DP705-02, TIANGEN, Beijing, China). Subsequently, genotyping for the SNP rs864745 was carried out using the SNaPshot Multiplex System on a genetic analyzer (3730XL, Applied Biosystems, Waltham, MA, USA). The resulting electropherograms were scrutinized with precision using the GeneMapper ID-X software version 4.0 (Thermo Fisher Scientific, Waltham, MA, USA), ensuring the accuracy and reliability of the genetic data obtained.

### 2.5. Identification of Glucose Metabolism and Calculation of HOMA2

Elevated fasting glucose was characterized by a fasting glucose level of ≥6.1 mmol/L or as the state of being under glucose-lowering pharmacotherapy [24]. The homeostasis model assessment of insulin resistance (HOMA2-IR) and the homeostasis model assessment of β-cell function (HOMA2-β) were computed utilizing the HOMA2 Calculator version 2.2.3, accessible via the website of the Oxford Centre for Diabetes, Endocrinology, and Metabolism (www.dtu.ox.ac.uk, accessed on 20 February 2024) [25].

### 2.6. Potential Confounders

Potential confounders were collected through an interviewer-led questionnaire that captured individuals’ age, sex, body mass index (BMI), household income (determined by dividing the aggregate annual family income by the total count of family members), education level (measured in years of education), dietary energy, intentional physical exercise (designated as bodily workouts aimed at preserving wellness and physical fitness), smoking status (never, former, and current), and alcohol consumption (classified into abstainers, social drinkers, infrequent heavy drinkers, and frequent heavy drinkers).

### 2.7. Statistical Analysis

Using logistic regression models to analyze the odds ratios (ORs) and 95% confidence intervals (CIs) for elevated fasting glucose, with the occurrence of elevated fasting glucose serving as the dependent variable. The independent variables included a product term comprising the presence of at least one C allele on rs864745 (a binary variable, coded as 1 for presence and 0 for absence) and the quartiles of dietary iron. After excluding 170 participants who were receiving glucose-lowering treatment, general linear regression was employed to evaluate the association between dependent variables (fasting glucose, HbA1c, and HOMA2 index, AST-to-ALT ratio) and independent variables (ferritin, dietary iron and the SNP rs864745), utilizing β coefficients and 95% CIs. The AST/ALT ratio was analyzed as a continuous variable, with detailed evaluations conducted in 0.1-unit increments to explore its correlation with dietary iron and the SNP rs864745. All analyses were performed using RGui, version 4.3.2 (http://www.r-project.org, accessed on 15 April 2023). Statistical significance was set at a two-tailed *p* < 0.05. Additionally, the ‘epiR’ package was employed to assess deviations from additivity in the interaction effects.

## 3. Results

### 3.1. Characteristics of the Participants

The participant demographics are summarized in Table 1, encompassing a comprehensive sample of 3298 Chinese adults, comprised 1584 males (48.0%) and 1714 females (52.0%). In terms of dietary habits, the average daily iron stood at 19.3 ± 15.6 mg across all participants, with a marginal variation between sex: 21.5 ± 19.3 mg for males and slightly lower at 17.3 ± 10.6 mg for females. Tables S1–S3 present the differences in characteristics among the four groups (Q1–Q4) classified based on dietary iron intake (Supplementary Material Tables S1–S3).

### 3.2. Genotypes of the JAZF1 rs864745

The prevalence of rs864745 genotypes across the participant pool revealed a notable breakdown: 62.8% carried the TT genotype, 32.2% harbored the TC genotype, and a minority of 4.9% exhibited the CC genotype, as outlined in Table 2. Furthermore, the MAF of the C allele stood at 21.1%, which was 21.0% among the males and slightly elevated to 21.1% among the females.

### 3.3. The Associations of Elevated Fasting Glucose with Dietary Iron and the JAZF1 rs864745

#### 3.3.1. Associations Between Dietary Iron, the *JAZF1* rs864745 Site, and Risk of Elevated Fasting Glucose

After accounting for various factors such as age, sex, BMI, income, education, intentional physical exercise, smoking status, alcohol use, and total dietary energy, our analysis revealed a significant positive correlation between dietary iron and the risk of elevated fasting glucose levels (*p* = 0.012), whereas no such association was observed for the SNP rs864745 (*p* = 0.932) across all participants.

When we segregated the data by sex and adjusted for the same confounding variables except sex, both dietary iron and the SNP rs864745 emerged as being linearly associated with increased risk of elevated fasting glucose, specifically among the male subjects (*p* = 0.006 and *p* = 0.047, respectively). Compared with the subgroup in the lowest quartile of dietary iron intake Q1 (<14.07 mg/day), the ORs (95% CI) for the elevated fasting glucose were 1.52 (1.01, 2.45) in the second quartile (14.07–17.79 mg/day), 1.73 (1.05, 3.00) in the third quartile (17.79–23.59 mg/day), and 2.49 (1.33, 4.74) in the highest quartile (≥23.59 mg/day). Notably, among the males, we also identified a multiplicative interaction between the SNP rs864745 and dietary iron, suggesting a combined effect on this risk (*p =* 0.041). Conversely, in the female participants, neither dietary iron nor the SNP rs864745 showed any significant association with elevated fasting glucose risk (*p* = 0.999 and *p* = 0.131, respectively), and no multiplicative interaction was detected between these two factors (*p* = 0.302). These findings are summarized in Table 3. We also scrutinized the outcomes derived from both recessive and codominant models and concurrently conducted assessments for the HOMA2 index, elevated fasting insulin, and fasting glycated hemoglobin; both yielded negative results (Supplementary File S1 Tables S3–S6).

#### 3.3.2. Associations Between Dietary Iron and Risk of Elevated Fasting Glucose Stratified by *JAZF1* rs864745

**Figure 2:**
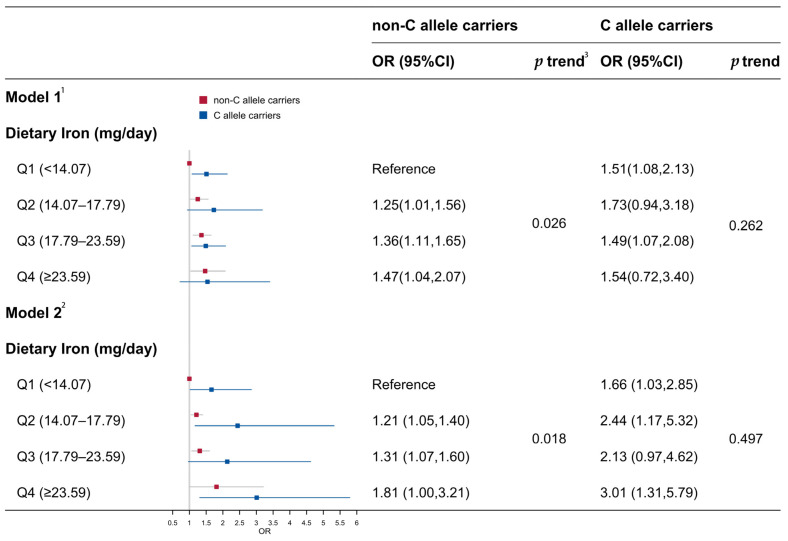
The association between dietary iron and risk of elevated fasting glucose stratified by C allele presence of the SNP rs864745 among the male participants. ^1^ Model 1 was adjusted for age. ^2^ Model 2 was adjusted for age, income, BMI, education, intentional physical exercise, smoking status, alcohol use, and total dietary energy. ^3^ The *p*-value for the trend was examined using the medians in each quartile of dietary iron.

Given the exclusive observation of the associations in the male participants and the significant *p*-value discovered in the preceding interaction analysis indicating the presence of heterogeneity within subgroups, a subsequent analysis was, thus, focused solely on the males to further stratify and investigate this heterogeneity. Within this framework, the C allele on rs864745 was designated as the risk allele. Upon adjustment for confounders such as age, income, BMI, educational years, intentional exercise routines, smoking habits, alcohol consumption, and dietary energy (Model 2), a notable trend persisted between dietary iron and the risk of elevated fasting glucose among individuals devoid of the C allele (*p* = 0.018). Conversely, this relationship was absent in those carrying the C allele (*p* = 0.487). Comparing the ORs across quartiles of dietary iron against the reference group (lowest intake subgroup among non-C allele carriers), the ORs for elevated fasting glucose risk were found to be 1.66 (1.03,2.85), 2.44 (1.17,5.32), 2.13 (0.97,4.62), and 3.01 (1.31,5.79), respectively (Figure 2).

### 3.4. The Association of Liver Metabolic Indicators with Ferritin and the JAZF1 rs864745

Using male participant data for analysis, after adjusting for the same covariates as in previous studies except for sex, it was found that both ferritin and the SNP rs864745 were associated with the AST-to-ALT ratio (*p* = 0.005 and *p* = 0.048, respectively) (Table 4).

## 4. Discussion

Consistent with previous findings, our study had revealed a positive correlation between the SNP rs864745, dietary iron, and elevated fasting glucose among the male participants, whereas no significant association was observed in females. Dietary iron overload can lead to iron overload (IO) in the body. As a potent oxidant, excessive free iron had significantly promoted the generation of reactive oxygen species, thereby inducing organ-specific oxidative stress [26], causing liver damage, disrupting lipid metabolism, and ultimately potentially leading to IR [27,28]. Research had indicated that even mild iron overload resulting from excessive iron through daily diet was a potential risk factor for both T2D and gestational diabetes mellitus (GDM) [29]. Given the fact that over one-tenth of adults in China currently suffered from IO, the rationality of appropriate dietary iron range deserves increased attention. According to the China Health and Nutrition Survey (CHNS) data, the prevalence of IO was lower among females compared to males [30], suggesting that the unique periodic blood loss experienced by women may serve as a protective mechanism against excessive iron accumulation in the body. This phenomenon explained why, in our study, we did not observe a significant association among the female participants.

Furthermore, among the male participants, we observed that the SNP rs864745 not only exhibits a significant association with elevated fasting glucose but also interacts with dietary iron. In line with previous findings in East Asian populations, the C allele was identified as the risk allele and differs from the European population [18,20,31]. Compared with the reference group (the subgroup with the lowest dietary iron and non-carriers of the C allele), individuals carrying the C allele exhibited a higher risk of elevated fasting glucose. A study conducted in the Chinese population had indicated that the C allele of rs864745 was associated with decreased insulin production and secretion, potentially underlying its contribution to abnormal fasting glucose levels [32]. We also examined the associations between fasting insulin levels, HOMA2 index, and both SNP s864745 and dietary iron, but no positive findings were observed. These results suggested the need for further extensive research into the relationship between rs864745 and insulin metabolism. Among the male participants without the C allele, a linear trend was observed between dietary iron and the risk of elevated fasting glucose, whereas no such trend was observed among carriers of the C allele. Over the past 40 years, there had been a notable decline in the proportion of plant-based foods and a remarkable increase in animal-based foods within the diets of Chinese residents, which had resulted in an elevated intake of heme iron sourced from animal-based foods [33]. Furthermore, the formulation of the recommended dietary iron in residents’ dietary guidelines had tended to prioritize meeting the minimum requirement for dietary iron, with the potential health risks associated with excessive iron being inadequately considered or incorporated. Compared to the European population (26.3%) [18], the Chinese population exhibited a significantly higher frequency of the TT (non-carriers of the C allele) genotype (62.5%), thereby emphasizing the critical necessity of regulating dietary iron, particularly among non-carriers of the C allele who are more susceptible to the impact of dietary iron and constitute the majority of the population.

To evaluate the potential relationship between liver function impairment caused by carrying the risk allele of rs864745 and body iron load, we assessed the correlation between the AST/ALT ratio, ferritin level, and the SNP rs864745. The results indicated a negative correlation between the AST/ALT ratio and ferritin level, as well as the presence of the C risk allele. The AST/ALT ratio was not only utilized in assessing hepatitis but also frequently in evaluating metabolic syndrome, serving as a pivotal indicator of liver function [34,35]. Notably, a decrease in the AST/ALT ratio was considered an important hallmark for non-alcoholic fatty liver disease (NAFLD) [36]. Studies conducted in East Asian cohorts had demonstrated a negative correlation between the AST/ALT ratio and the incidence of T2D [37]. The liver bore the responsibility of handling 60–65% of the glucose load [38], minimizing postprandial glucose fluctuations through absorption and storage of glucose, thereby playing a pivotal role in the pathogenesis of elevated fasting glucose [38]. Based on these observations, we hypothesize that the combined effect of ferritin and the SNP rs864745 may potentially influence elevated fasting glucose by modulating liver function.

It is essential to acknowledge the limitations inherent in our study. Firstly, the estimation of dietary energy and iron relied on a 3-day 24 h dietary recall method, which is subject to recall bias, potentially leading to biased estimates. This methodology inherently introduces subjectivity and may not fully capture the actual dietary patterns. Furthermore, our assessment of iron load was limited to ferritin data alone, without the inclusion of other serum markers such as free iron or transferrin. This limited scope prevented a comprehensive evaluation of the body’s iron status and load. And owing to the complexity encountered during field investigations, we were unable to perform the Oral Glucose Tolerance Test (OGTT), resulting in the absence of 2 h insulin data. Consequently, a comprehensive assessment of insulin function and sensitivity was not feasible. Last, it is crucial to note that our analyses were conducted using data from a cross-sectional study design. As such, we were unable to draw definitive causal inferences between the SNP rs864745, dietary iron, and elevated fasting glucose. Longitudinal or interventional studies would be necessary to establish a causal relationship between these variables.

## 5. Conclusions

Our study found that dietary iron and the SNP rs864745 were interacted and associated with elevated fasting glucose in Chinese males, absent in females. In addition, the presence of C allele on rs864745 showed higher risks of elevated fasting glucose in Chinese male population which constantly associated with high risks whether under low or high dietary irons. The SNP and iron load might affect liver function, impacting elevated fasting glucose.

## Figures and Tables

**Table 1:** Characteristics of the participants, stratified by sex.

	All	Male	Female
n (%)	3298 (100.0)	1584 (48.0)	1714 (52.0)
Age (%)			
15–44 years	972 (29.5)	458 (29.0)	514 (30.0)
45–59 years	1263 (38.3)	600 (37.9)	663 (38.7)
60 years	1059 (32.1)	524 (33.1)	535 (31.2)
Annual Household Income (%)			
Above average level (RMB > 60,000) ^1^	119 (4.5)	61 (4.9)	58 (4.2)
Average level (RMB 30,000–59,999)	1473 (56.2)	693 (55.9)	780 (56.5)
Below average level (RMB < 30,000)	810 (30.9)	391 (31.5)	419 (30.3)
No answer	219 (8.4)	95 (7.7)	124 (9.0)
Years of Education, years (SD) ^2^	9.5 (4.5)	10.1 (4.1)	8.9 (4.8)
Intentional Physical Exercise (%)			
Yes	2454 (74.8)	1184 (75.2)	1270 (74.4)
no	828 (25.2)	391 (24.8)	437 (25.6)
Smoking Status (%)			
Never smoked	2312 (70.2)	620 (39.2)	1692 (98.8)
Former smoker	177 (5.4)	171 (10.8)	6 (0.4)
Current smoker	805 (24.4)	790 (50.0)	15 (0.9)
Alcohol Use (%)			
Lifetime abstainers	2471 (79.3)	899 (61.6)	1572 (94.9)
Nonheavy drinkers	503 (16.1)	429 (29.4)	74 (4.5)
Infrequent heavy drinkers	45 (1.4)	41 (2.8)	4 (0.2)
Frequent heavy drinkers	96 (3.1)	90 (6.2)	6 (0.4)
BMI ^3^ (SD)	24.0 (5.0)	24.1 (5.7)	23.8 (3.5)
Dietary Intake			
Energy, kcal/day (SD)	1772.5 (890.5)	1957.2 (1002.1)	1601.8 (733.2)
Total iron, mg/day (SD)	19.3 (15.6)	21.5 (19.3)	17.3 (10.6)
Glucose Metabolism Index			
Elevated Fasting Glucose (%)			
Yes	442 (13.4)	242 (15.3)	200 (11.7)
No	2856 (86.6)	1342 (84.7)	1514 (88.3)
FPG ^4^, mmol/L (SD)	5.4 (1.6)	5.4 (1.6)	5.3 (1.5)
HbA1c ^5^, mmol/L (SD)	5.8 (1.2)	5.9 (1.3)	5.8 (1.1)
HOMA-β ^6^ (SD)	76.8 (91.5)	74.3 (96.8)	79.1 (86.5)
HOMA2-IR ^7^ (SD)	1.4 (1.9)	1.4 (2.4)	1.4 (1.5)
Liver Function Index			
ALT ^8^, mmol/L (SD)	20.1 (19.9)	22.4 (16.8)	18.0 (22.2)
AST ^9^, mmol/L (SD)	22.4 (11.6)	23.4 (10.5)	21.5 (12.4)
AST/ALT (SD)	1.3 (0.5)	1.2 (0.5)	1.4 (0.5)
Body Iron Load Index			
Ferritin, mmol/L (SD)	121.2(117.4)	160.6 (132.1)	85.7 (88.6)

^1^ RMB, renminbi; ^2^ SD, standard deviation; ^3^ BMI, Body Mass Index; ^4^ FPG, fasting plasma glucose; ^5^ HbA1c, Glycated Hemoglobin A1c; ^6^ HOMA2-IR, homeostasis model assessment of insulin resistance; ^7^ HOMA2-β, homeostasis model assessment of β-cell function; ^8^ ALT alanine aminotransferase; ^9^ AST aspartate aminotransferase.

**Table 2:** Genotypes of the *JAZF1* rs864745 in the study participants.

	Frequency (%)			
	All	Male	Female	*p* Value ^6^
Genotype				
C allele carriers ^1^				0.339
TC ^2^	849 (32.2)	376 (30.3)	470 (34.0)	
CC ^3^	130(4.9)	73 (5.9)	56 (4.1)	
C allele non-carriers				
TT ^4^	1655 (62.8)	792 (63.8)	856 (61.9)	
MAF ^5^				
C	21.1	21.0	21.1	1.000

^1^ Who at least had one C allele on the rs864745, including TC and CC; ^2^ TC, one T allele and one C allele; ^3^ CC, double C allele; ^4^ TT, double T allele; ^5^ MAF, minor allele frequency; ^6^ The *p*-value for testing whether there was a difference in the proportion of the C allele carriers and MAF of C between males and females was determined using the Chi-squared test.

**Table 3:** Odds ratios (ORs) (95% CI) and multiplicative interaction results for risk of elevated fasting glucose according to dietary iron and the SNP rs864745 in the participants stratified by sex ^1^.

	Model 1 ^2^			Model 2 ^3^		
	OR (95% CI) ^4^	*p*_trend_ ^5^	*p*_INTM_ ^6^	OR (95% CI)	*p*_trend_	*p*_INTM_
All						
Dietary iron			0.663			0.620
Q1 (<12.63 mg/day)	Reference	0.008		Reference	0.012	
Q2 (12.63–16.53 mg/day)	0.93 (0.64, 1.36)			0.98 (0.65, 1.46)		
Q3 (16.53–21.62 mg/day)	1.08 (0.75, 1.55)			1.18 (1.00, 1.69)		
Q4 (≥21.62 mg/day)	1.27 (0.89, 1.81)			1.73 (1.11, 2.72)		
rs864745						
Non-C allele carriers	Reference	0.983		Reference	0.932	
C allele carriers	0.99 (0.58, 1.68)			1.05 (0.60, 1.81)		
Male						
Dietary iron			0.043			0.041
Q1 (<14.07 mg/day)	Reference	0.046		Reference	0.006	
Q2 (14.07–17.79 mg/day)	1.18 (0.65, 2.19)			1.52 (1.01, 2.45)		
Q3 (17.79–23.59 mg/day)	1.52 (1.07, 1.73)			1.73 (1.05, 3.00)		
Q4 (≥23.59 mg/day)	1.75 (1.03, 3.07)			2.49 (1.33, 4.74)		
rs864745						
Non-C allele carriers	Reference	0.049		Reference	0.047	
C allele carriers	1.95 (1.01, 3.93)			2.15 (1.02, 4.51)		
Female						
Dietary iron			0.186			0.302
Q1 (<11.61 mg/day)	Reference	0.974		Reference	0.999	
Q2 (11.61–16.00 mg/day)	0.85 (0.52, 1.39)			0.71 (0.40, 1.24)		
Q3 (16.00–19.87 mg/day)	0.86 (0.52, 1.41)			0.80 (0.46, 1.40)		
Q4 (≥19.87 mg/day)	1.04 (0.61, 1.74)			0.98 (0.53, 1.85)		
rs864745						
Non-C allele carriers	Reference	0.068		Reference	0.131	
C allele carriers	0.49 (0.23, 1.04)			0.52 (0.26, 0.98)		

^1^ C allele presence of rs864745 was coded as 0 for non-presence and 1 for presence. Dietary iron was categorized into four groups based on quartiles, each including its lower boundary, with Q1 serving as the reference. ^2^ Model 1 was adjusted for age. ^3^ Model 2 was adjusted for age, sex, income, BMI, education, intentional physical exercise, smoking status, alcohol use, and total dietary energy. ^4^ OR (95% CI) for Dietary Iron represents the risk of elevated fasting glucose occurrence of the current dietary iron range compared with the reference group. OR (95% CI) for rs864745 represented the multiplicative increase in the risk of Elevated Fasting Glucose for individuals carrying the C allele, compared to those who do not carry the C allele. ^5^ *p*_trend_, the *p* value for the trend was examined using the medians in each quartile of dietary iron. ^6^ *p*_INTM_, the *p* value of multiplicative interaction.

**Table 4:** General linear regression results for the AST-to-ALT ratio according to ferritin level and the SNP rs864745 in the male participants ^1^.

	Β ^2^	*p* Value
Ferritin	−0.0004	0.005
rs864745	−0.017	0.048

^1^ C allele presence of rs864745 was coded as 1 for presence and 0 for non-presence, ferritin was included as a continuous variable. ^2^ β serves as the partial correlation coefficient in our models, reflecting the AST-to-ALT ratio changes in response to either a one-unit fluctuation (mmol/L) in ferritin levels or the presence of the C allele on rs864745, compared with the reference group.

## Data Availability

The datasets used and analyzed during the current study are available from the corresponding author upon reasonable request.

## Supplementary Materials

The following supporting information can be downloaded at: https://www.mdpi.com/article/10.3390/nu16223831/s1, Table S1: Characteristics of the participants, stratified by dietary iron. Table S2: Characteristics of the male participants, stratified by dietary iron. Table S3: Characteristics of the female participants, stratified by dietary iron. Table S4: Odds Ratios (ORs) with 95% Confidence Intervals (CIs) and Multiplicative Interaction Results for Elevated Risk of HOMA2 Index, Elevated Fasting Insulin and Fasting Glycated Hemoglobin, Based on Dietary Iron Intake and SNP rs864745, in Participants Stratified by Gender, Under a Dominant Inheritance Model. Table S5: Odds Ratios (ORs) with 95% Confidence Intervals (CIs) and Multiplicative Interaction Results for Elevated Risk of HOMA2 Index, Elevated Fasting Insulin and Fasting Glycated Hemoglobin, Based on Dietary Iron Intake and SNP rs864745, in Participants Stratified by Gender, Under Recessive Inheritance Model. Table S6: Odds Ratios (ORs) with 95% Confidence Intervals (CIs) and Multiplicative Interaction Results for Elevated Risk of HOMA2 Index, Elevated Fasting Insulin and Fasting Glycated Hemoglobin, Based on Dietary Iron Intake and SNP rs864745, in Participants Stratified by Gender, Under a Codominant Inheritance Model. Table S7: Odds ratios (ORs) (95% CI) and multiplicative interaction results for risk of elevated fasting glucose according to dietary iron and the SNP rs864745 in the participants stratified by sex.

## Abbreviations

IDF: International Diabetes Federation; CCDRFS, China Chronic Disease and Risk Factors Surveillance; ADA, American Diabetes Association; IR, insulin resistance; T2D, type 2 diabetes; JAZF1, JAZF Zinc Finger 1; ER, endoplasmic reticulum; SNP, single nucleotide polymorphisms; GWAS, Genome-wide association studies; MAF, minor allele frequency; FFQ, food frequency questionnaire; RMB, renminbi; SD, standard deviation. FPG, fasting plasma glucose; HbA1c, Glycated Hemoglobin A1c; HOMA2-IR, homeostasis model assessment of insulin resistance HOMA2-β, homeostasis model assessment of β-cell function; ALT alanine aminotransferase; AST aspartate aminotransferase; OR, odds ratio; 95% CI, 95% confidence interval; RERI, Relative Excess Risk due to Interaction; AP, Attributable Proportion; S, Synergy index; IO, iron overload; CHNS, China Health and Nutrition Survey; NAFLD, non-alcoholic fatty liver disease.
