# Supplementary material for: The Association Between Dietary Iron, the SNP of the JAZF1 rs864745, and Glucose Metabolism in a Chinese Population
Source: Nutrients. 2024 Nov 8;16(22):3831. doi: 10.3390/nu16223831 (PMC11597416; doi:10.3390/nu16223831)
Supplement: Supplementary file 1 [file nutrients-16-03831-s001.zip › nutrients-3277226-supplementary.pdf]

Table S1. Characteristics of the participants, stratified by dietary iron

	All	Q1	Q2	Q3	Q4	*p* value
n (%)	3298(100.0)	1584(48.0)	1714(52.0)	1584(48.0)	1584(48.0)	
Age (%)						
15−44 years	972(29.5)	227(27.5)	273(33.1)	237(28.9)	235(28.5)	
45–59 years	1263(38.3)	292(35.4)	310(37.6)	327(39.8)	334(40.5)	0.008
60− years	1059(32.1)	305(37.0)	241(29.2)	257(31.3)	256(31.0)	
Annual Household Income (%)						
Above average level (RMB > 60,000)[1]	147(4.5)	36(4.4)	35(4.2)	46(5.6)	30(3.6)	
Average level (RMB 30,000–59,999)	1870(56.8)	523(63.5)	513(62.3)	433(52.7)	401(48.6)	<0.001
Below average level (RMB < 30,000)	1024(31.1)	212(25.7)	215(26.1)	284(34.5)	313(37.9)	
No answer	254(7.7)	53(6.4)	61(7.4)	59(7.2)	81(9.8)	
Years of Education, years (SD)[2]	9.5(4.5)	8.1(4.9)	9.4(4.4)	9.9(4.2)	10.5(3.9)	<0.001
Intentional Physical Exercise (%)						
Yes	2454(74.8)	673(82.2)	634(77.0)	577(70.5)	570(69.3)	<0.001
no	828(25.2)	146(17.8)	189(23.0)	241(29.5)	252(30.7)	
Smoking Status (%)						
Never smoked	2312(70.2)	670(81.3)	589(71.5)	555(67.5)	498(60.4)	
Former smoker	177(5.4)	32(3.9)	45(5.5)	45(5.5)	55(6.7)	<0.001
Current smoker	805(24.4)	122(14.8)	190(23.1)	222(27.0)	271(32.9)	
Alcohol Use (%)						
Lifetime abstainers	2471(79.3)	675(86.5)	638(81.1)	608(77.7)	550(71.9)	
Nonheavy drinkers	503(16.1)	84(10.8)	105(13.3)	136(17.4)	178(23.3)	<0.001
Infrequent heavy drinkers	45(1.4)	7(0.9)	15(1.9)	12(1.5)	11(1.4)	
Frequent heavy drinkers	96(3.1)	14(1.8)	29(3.7)	27(3.4)	26(3.4)	
BMI[3] (SD)	24.0(5.0)	23.5(3.4)	24.1(7.8)	24.2(3.7)	24.1(3.6)	0.011
Dietary Intake						
Energy, kcal/day (SD)	1772.5(890.5)	1118.5(323.2)	1554.8(366.9)	1860.6(434.1)	2548.9(1286.9)	<0.001
Total iron, mg/day (SD)	19.3(15.6)	9.9(2.2)	14.6(1.1)	18.8(1.5)	33.9(25.2)	<0.001
Glucose Metabolism Index						
Elevated Fasting Glucose (%)						
Yes	2856(86.6)	726(88.0)	717(87.0)	709(86.0)	704(85.3)	0.411
No	442(13.4)	99(12.0)	107(13.0)	115(14.0)	121(14.7)	
FPG[4], mmol/L (SD)	5.4(1.6)	5.3(1.5)	5.3(1.3)	5.4(1.9)	5.4(1.5)	0.316
HbA1c[5], mmol/L (SD)	5.8(1.2)	5.8(1.1)	5.8(1.2)	5.9(1.3)	5.9(1.2)	0.355
HOMA-β[6] (SD)	76.8(91.5)	76.1(89.7)	79.7(104.8)	80.0(86.8)	71.6(83.2)	0.308
HOMA2-IR[7] (SD)	1.4(1.9)	1.3(1.9)	1.3(1.6)	1.4(2.2)	1.4(2.0)	0.526
Insulin (SD)	5.6(7.0)	5.6(7.4)	5.3(5.3)	5.8(7.5)	5.6(7.4)	0.547

Liver Function Index						
ALT[8], mmol/L (SD)	20.1(19.9)	18.6(14.3)	19.5(26.2)	21.3(21.2)	21.1(15.7)	0.018
AST[9], mmol/L (SD)	22.4(11.6)	22.0(9.9)	22.3(12.3)	22.7(13.7)	22.5(9.9)	0.656
AST/ALT (SD)	1.3(0.5)	1.4(0.5)	1.4(0.5)	1.3(0.5)	1.3(0.5)	<0.001
Body Iron Load Index						
Ferritin, mmol/L (SD)	121.2(117.4)	109.0(127.3)	118.1(106.7)	126.5(119.7)	130.6(114.5)	0.004

[1] RMB, renminbi; [2] SD, standard deviation. [3] BMI, Body Mass Index [4] FPG, fasting plasma glucose; [5] HbA1c, Glycated Hemoglobin A1c; [6] HOMA2-IR, homeostasis model assessment of insulin resistance [7] HOMA2-β, homeostasis model assessment of β-cell function; [8] ALT alanine aminotransferase; [9] AST aspartate aminotransferase.

Table S2 Characteristics of the male participants, stratified by dietary iron

	**Male**	**Q1**	**Q2**	**Q3**	**Q4**	***p* value**
n (%)	3298(100.0)	1584(48.0)	1714(52.0)	1584(48.0)	1584(48.0)	
Age (%)						
15−44 years	458(29.0)	113(28.5)	125(31.7)	110(27.8)	110(27.8)	
45–59 years	600(37.9)	131(33.0)	158(40.1)	154(39.0)	157(39.6)	0.078
60− years	524(33.1)	153(38.5)	111(28.2)	131(33.2)	129(32.6)	
Annual Household Income (%)						
Above average level (RMB > 60,000)[1]	71(4.5)	17(4.3)	17(4.3)	25(6.3)	12(3.0)	
Average level (RMB 30,000–59,999)	902(57.0)	261(65.7)	240(60.9)	219(55.4)	182(46.0)	<0.001
Below average level (RMB < 30,000)	495(31.3)	95(23.9)	106(26.9)	133(33.7)	161(40.7)	
No answer	114(7.2)	24(6.0)	31(7.9)	18(4.6)	41(10.4)	
Years of Education, years (SD)[2]	10.1(4.0)	9.1(4.2)	10.1(4.1)	10.5(3.8)	10.8(3.7)	<0.001
Intentional Physical Exercise (%)						
Yes	1184(75.2)	331(83.8)	295(75.1)	275(70.0)	283(71.8)	<0.001
no	391(24.8)	64(16.2)	98(24.9)	118(30.0)	111(28.2)	
Smoking Status (%)						
Never smoked	620(39.2)	184(46.3)	157(39.8)	134(33.9)	145(36.7)	
Former smoker	171(10.8)	45(11.3)	39(9.9)	46(11.6)	41(10.4)	0.001
Current smoker	790(50.0)	168(42.3)	198(50.3)	215(54.4)	209(52.9)	
Alcohol Use (%)						
Lifetime abstainers	899(61.6)	251(68.6)	218(59.7)	223(60.1)	207(58.0)	
Nonheavy drinkers	429(29.4)	88(24.0)	99(27.1)	120(32.3)	122(34.2)	0.019
Infrequent heavy drinkers	41(2.8)	7(1.9)	17(4.7)	9(2.4)	8(2.2)	
Frequent heavy drinkers	90(6.2)	20(5.5)	31(8.5)	19(5.1)	20(5.6)	
BMI[3] (SD)	24.2(3.3)	23.6(3.3)	24.3(3.4)	24.5(3.3)	24.4(3.3)	0.008
Dietary Intake						
Energy, kcal/day (SD)	1957.2(1002.1)	1286.3(347.0)	1710.0(405.7)	2056.2(458.6)	2770.5(1529.1)	<0.001
Total iron, mg/day (SD)	21.5(19.3)	11.2(2.2)	16.0(1.1)	20.4(1.7)	38.4(32.6)	<0.001
Glucose Metabolism Index						

Elevated Fasting Glucose (%)						
Yes	1342(84.7)	342(86.1)	332(84.1)	336(84.8)	332(83.8)	0.800
No	242(15.3)	55(13.9)	63(15.9)	60(15.2)	64(16.2)	
FPG[4], mmol/L (SD)	5.4(1.6)	5.4(1.5)	5.4(2.0)	5.4(1.6)	5.4(1.5)	0.900
HbA1c[5], mmol/L (SD)	5.9(1.3)	5.9(1.3)	5.8(1.2)	5.9(1.4)	5.9(1.2)	0.643
HOMA-β[6] (SD)	74.3(96.8)	73.9(102.8)	74.0(77.5)	74.6(96.4)	74.7(108.5)	0.999
HOMA2-IR[7] (SD)	1.4(2.4)	1.3(2.2)	1.3(2.3)	1.4(2.4)	1.4(2.5)	0.910
Insulin (SD)	5.5(8.5)	5.3(8.7)	5.3(7.2)	5.6(8.3)	5.7(9.7)	0.913
Liver Function Index						
ALT[8], mmol/L (SD)	22.4(16.8)	21.9(16.0)	22.1(17.0)	23.1(19.5)	22.7(14.6)	0.753
AST[9], mmol/L (SD)	23.4(10.5)	23.2(9.3)	23.6(10.4)	23.3(12.4)	23.2(9.5)	0.945
AST/ALT (SD)	1.2(0.5)	1.3(0.5)	1.3(0.5)	1.2(0.5)	1.2(0.5)	0.041
Body Iron Load Index						
Ferritin, mmol/L (SD)	160.6(132.1)	160.1(148.5)	155.0(116.2)	159.1(138.6)	168.3(124.1)	0.646

[1] RMB, renminbi; [2] SD, standard deviation. [3] BMI, Body Mass Index [4] FPG, fasting plasma glucose; [5] HbA1c, Glycated Hemoglobin A1c; [6] HOMA2-IR, homeostasis model assessment of insulin resistance [7] HOMA2-β, homeostasis model assessment of β-cell function; [8] ALT alanine aminotransferase; [9] AST aspartate aminotransferase.

Table S3 Characteristics of the female participants, stratified by dietary iron

	Female	Q1	Q2	Q3	Q4	*p* value
n (%)	3298(100.0)	1584(48.0)	1714(52.0)	1584(48.0)	1584(48.0)	
Age (%)						
15−44 years	514(30.0)	125(29.2)	139(32.5)	126(29.5)	124(28.9)	
45–59 years	663(38.7)	147(34.3)	170(39.7)	160(37.5)	186(43.4)	0.037
60− years	535(31.2)	156(36.4)	119(27.8)	141(33.0)	119(27.7)	
Annual Household Income (%)						
Above average level (RMB > 60,000)[1]	76(4.4)	17(4.0)	20(4.7)	22(5.1)	17(4.0)	
Average level (RMB 30,000–59,999)	968(56.5)	282(65.9)	248(57.9)	238(55.6)	200(46.6)	<0.001
Below average level (RMB < 30,000)	529(30.9)	108(25.2)	117(27.3)	136(31.8)	168(39.2)	
No answer	140(8.2)	21(4.9)	43(10.0)	32(7.5)	44(10.3)	
Years of Education, years (SD)[2]	8.9(4.8)	7.4(5.2)	8.9(5.0)	9.1(4.5)	10.1(4.1)	<0.001
Intentional Physical Exercise (%)						
Yes	1270(74.4)	342(80.3)	327(76.8)	312(73.1)	289(67.5)	0.001
no	437(25.6)	84(19.7)	99(23.2)	115(26.9)	139(32.5)	
Smoking Status (%)						
Never smoked	1692(98.8)	422(98.6)	424(99.1)	423(98.8)	423(98.6)	
Former smoker	6(0.4)	1(0.2)	1(0.2)	1(0.2)	3(0.7)	0.913
Current smoker	15(0.9)	5(1.2)	3(0.7)	4(0.9)	3(0.7)	
Alcohol Use (%)						
Lifetime abstainers	1572(94.9)	393(95.4)	401(96.4)	395(94.7)	383(93.2)	0.197

Nonheavy drinkers	74(4.5)	17(4.1)	14(3.4)	18(4.3)	25(6.1)	
Infrequent heavy drinkers	4(0.2)	1(0.2)	0(0.0)	2(0.5)	1(0.2)	
Frequent heavy drinkers	6(0.4)	1(0.2)	1(0.2)	2(0.5)	2(0.5)	
BMI[3] (SD)	23.8(6.1)	23.5(3.5)	24.1(10.3)	23.8(3.7)	23.8(4.3)	0.522
Dietary Intake						
Energy, kcal/day (SD)	1601.8(733.2)	1031.8(311.2)	1420.2(357.2)	1679.0(385.7)	2269.4(983.0)	<0.001
Total iron, mg/day (SD)	17.3(10.6)	9.1(2.0)	13.4(1.0)	17.3(1.3)	29.4(14.6)	<0.001
Glucose Metabolism Index						
Elevated Fasting Glucose (%)						
Yes	1514(88.3)	376(87.6)	384(89.7)	378(88.3)	376(87.6)	0.755
No	200(11.7)	53(12.4)	44(10.3)	50(11.7)	53(12.4)	
FPG[4], mmol/L (SD)	5.3(1.5)	5.4(1.7)	5.2(1.1)	5.3(1.6)	5.4(1.5)	0.142
HbA1c[5], mmol/L (SD)	5.8(1.1)	5.8(1.2)	5.7(1.0)	5.9(1.1)	5.9(1.1)	0.272
HOMA-β[6] (SD)	79.1(86.5)	77.5(85.8)	83.8(78.4)	82.7(116.8)	72.2(53.1)	0.261
HOMA2-IR[7] (SD)	1.4(1.5)	1.4(1.7)	1.3(1.1)	1.4(1.4)	1.4(1.6)	0.502
Insulin (SD)	5.6(5.2)	5.8(6.5)	5.4(4.1)	5.6(4.8)	5.7(5.2)	0.836
Liver Function Index						
ALT[8], mmol/L (SD)	18.0(22.2)	16.5(12.7)	17.1(11.8)	19.9(37.9)	18.4(15.3)	0.105
AST[9,] mmol/L (SD)	21.5(12.4)	21.3(10.3)	21.1(9.0)	22.3(19.0)	21.4(8.3)	0.493
AST/ALT (SD)	1.4(0.5)	1.5(0.5)	1.4(0.4)	1.4(0.5)	1.4(0.5)	0.007
Body Iron Load Index						
Ferritin, mmol/L (SD)	85.7(88.6)	87.4(111.5)	82.5(76.9)	90.8(79.9)	82.2(82.7)	0.517

[1] RMB, renminbi; [2] SD, standard deviation. [3] BMI, Body Mass Index [4] FPG, fasting plasma glucose; [5] HbA1c, Glycated Hemoglobin A1c; [6] HOMA2-IR, homeostasis model assessment of insulin resistance [7] HOMA2-β, homeostasis model assessment of β-cell function; [8] ALT alanine aminotransferase; [9] AST aspartate aminotransferase.

Table S4. Odds Ratios (ORs) with 95% Confidence Intervals (CIs) and Multiplicative Interaction Results for Elevated Risk of HOMA2 Index, Elevated Fasting Insulin and Fasting Glycated Hemoglobin, Based on Dietary Iron Intake and SNP rs864745, in Participants Stratified by Gender, Under a Dominant Inheritance Model[1].

	Model 1[2]			Model 2[3]		
	**OR (95% CI)[4]**	p_{trend}[5]	p_{INTM}[6]	**OR (95% CI)**	p_{trend}	p_{INTM}
Elevated fasting glucose						
All						
Dietary iron						
Q1 (<12.63)	Reference			Reference		
Q2 (12.63-16.53)	0.93 (0.64,1.36)	0.008		1.03 (0.69,1.53)	0.004	
Q3 (16.53-21.62)	1.08 (0.75,1.55)		0.663	1.30 (1.10,1.65)		0.527
Q4 (≥21.62)	1.27 (0.89,1.81)			1.85 (1.19,2.89)		
rs864745						
C allele Non-Carriers	Reference	0.983		Reference	0.867	
C allele Carriers	0.99 (0.58,1.68)			1.05 (0.60,1.81)		
Male						
Dietary iron						
Q1 (<14.07)	Reference			Reference		
Q2 (14.07-17.79)	1.18 (0.65,2.19)	0.046		1.45 (1.01,2.12)	0.003	
Q3 (17.79-23.59)	1.52 (1.07,1.73)		0.043	2.20 (1.17,4.29)		0.027
Q4 (≥23.59)	1.75 (1.03,3.07)			3.12 (1.61,6.29)		
rs864745						
C allele Non-Carriers	Reference	0.049		Reference	0.042	
C allele Carriers	1.95 (1.01,3.93)			2.15 (1.02,4.51)		
Female						
Dietary iron						
Q1 (<11.61)	Reference			Reference		
Q2 (11.61-16.00)	0.85 (0.52,1.39)	0.974		0.87 (0.51,1.46)	0.613	
Q3 (16.00-19.87)	0.86 (0.52,1.41)		0.186	0.93 (0.54,1.60)		0.197
Q4 (≥19.87)	1.04 (0.61,1.74)			1.25 (0.65,2.39)		
rs864745						
C allele Non-Carriers	Reference	0.068		Reference	0.081	
C allele Carriers	0.49 (0.23,1.04)			0.51 (0.23,1.07)		
HbA1c						
All						
Dietary iron (mg/day)						
Q1 (<12.63)	Reference	0.774	0.982	Reference	0.33	0.9
Q2 (12.63-16.53)	1.09(0.83,1.43)			1.12(0.84,1.49)		

Q3 (16.53-21.62)	1.18(0.90,1.54)			1.26(0.94,1.70)		
Q4 (≥21.62)	1.14(0.87,1.48)			1.27(0.91,1.77)		
rs864745						
Non-C allele carriers	Reference	0.662		Reference	0.608	
C allele carriers	0.99(0.58,1.68)			1.05(0.60,1.81)		
Male						
Dietary iron (mg/day)						
Q1 (<14.07)	Reference			Reference		
Q2 (14.07-17.79)	1.04(0.67,1.61)	0.831		1.08(0.68,1.73)	0.317	
Q3 (17.79-23.59)	1.18(0.77,1.81)		0.595	1.28(0.80,2.05)		0.716
Q4 (≥23.59)	1.31(0.87,1.97)			1.52(0.93,2.50)		
rs864745						
C allele Non-Carriers	Reference	0.996		Reference	0.947	
C allele Carriers	1.95(1.01,3.93)			2.15(1.02,4.51)		
Female						
Dietary iron (mg/day)						
Q1 (<11.61)	Reference			Reference		
Q2 (11.61-16.00)	1.08(0.68,1.73)	0.799		1.16(0.80,1.69)	0.941	
Q3 (16.00-19.87)	1.28(0.80,2.05)		0.525	1.28(0.87,1.89)		0.478
Q4 (≥19.87)	1.52(0.93,2.50)			0.99(0.61,1.58)		
rs864745						
Non-C allele carriers	Reference	0.475		Reference	0.382	
C allele carriers	0.49(0.23,1.04)			0.51(0.23,1.07)		
HOMA-β						
All						
Dietary iron (mg/day)						
Q1 (<12.63)	Reference			Reference		
Q2 (12.63-16.53)	0.99(0.72,1.38)	0.119		1.06(0.74,1.51)	0.627	
Q3 (16.53-21.62)	1.02(0.74,1.42)		0.332	1.07(0.74,1.55)		0.49
Q4 (≥21.62)	0.87(0.64,1.20)			0.92(0.62,1.37)		
rs864745						
C allele Non-Carriers	Reference	0.315		Reference	0.388	
C allele Carriers	0.99(0.58,1.68)			1.05(0.60,1.81)		
Male						
Dietary iron (mg/day)						
Q1 (<14.07)	Reference			Reference		
Q2 (14.07-17.79)	0.78(0.46,1.29)	0.127	0.265	0.71(0.41,1.23)	0.429	0.321
Q3 (17.79-23.59)	0.98(0.59,1.60)			0.87(0.50,1.52)		
Q4 (≥23.59)	0.77(0.48,1.22)			0.65(0.37,1.15)		

rs864745						
Non-C allele carriers	Reference	0.204		Reference	0.216	
C allele carriers	1.95(1.01,3.93)			2.15(1.02,4.51)		
Female						
Dietary iron (mg/day)						
Q1 (<11.61)	Reference			Reference		
Q2 (11.61-16.00)	1.36(0.87,2.15)	0.051		1.32(0.82,2.15)	0.177	
Q3 (16.00-19.87)	1.24(0.79,1.98)		0.271	1.06(0.64,1.77)		0.341
Q4 (≥19.87)	1.66(0.99,2.88)			1.31(0.69,2.51)		
rs864745						
C allele Non-Carriers	Reference	0.387		Reference	0.476	
C allele Carriers	0.49(0.23,1.04)			1.69(0.99,2.96)		
HOMA2-IR						
All						
Dietary iron (mg/day)						
Q1 (<12.63)	Reference			Reference		
Q2 (12.63-16.53)	0.79(0.47,1.32)	0.802		0.90(0.52,1.57)	0.569	
Q3 (16.53-21.62)	1.04(0.64,1.68)		0.900	1.23(0.71,2.15)		0.602
Q4 (≥21.62)	1.08(0.67,1.74)			1.34(0.72,2.50)		
rs864745						
C allele Non-Carriers	Reference	0.515		Reference	0.735	
C allele Carriers	0.99(0.58,1.68)			1.05(0.60,1.81)		
Male						
Dietary iron (mg/day)						
Q1 (<14.07)	Reference			Reference		
Q2 (14.07-17.79)	0.86(0.37,2.04)	0.165		1.28(0.50,3.45)	0.061	
Q3 (17.79-23.59)	1.14(0.53,2.54)		0.662	1.97(0.79,5.29)		0.419
Q4 (≥23.59)	1.09(0.53,2.38)			1.93(0.73,5.51)		
rs864745						
C allele Non-Carriers	Reference	0.497		Reference	0.952	
C allele Carriers	1.95(1.01,3.93)			2.15(1.02,4.51)		
Female						
Dietary iron (mg/day)						
Q1 (<11.61)	Reference			Reference		
Q2 (11.61-16.00)	0.75(0.37,1.44)	0.514		0.75(0.37,1.52)	0.068	
Q3 (16.00-19.87)	0.96(0.49,1.81)		0.698	1.00(0.48,2.03)		0.808
Q4 (≥19.87)	1.12(0.56,2.17)			1.21(0.51,2.84)		
Dietary iron (mg/day)						
C allele Non-Carriers	Reference	0.636		Reference	0.616	
C allele Carriers	0.49(0.23,1.04)			0.51(0.23,1.07)		
Insulin						
All						

Dietary iron (mg/day)						
Q1 (<12.63)	Reference	0.802	0.851	Reference	0.569	0.995
Q2 (12.63-16.53)	0.18 (0.03,0.69)			0.19 (0.03,0.74)		
Q3 (16.53-21.62)	0.83 (0.33,2.06)			0.79 (0.29,2.15)		
Q4 (≥21.62)	0.36 (0.10,1.09)			0.29 (0.07,1.09)		
rs864745						
C allele Non-Carriers	Reference	0.515		Reference	0.735	
C allele Carriers	0.16 (0.01,0.80)			0.18 (0.01,0.94)		
Male						
Dietary iron (mg/day)						
Q1 (<14.07)	Reference	0.165	0.771	Reference	0.061	0.974
Q2 (14.07-17.79)	0.92 (0.17,5.06)			1.77 (0.27,14.24)		
Q3 (17.79-23.59)	0.93 (0.17,5.07)			1.80 (0.25,16.02)		
Q4 (≥23.59)	0.85 (0.16,4.64)			1.36 (0.16,13.80)		
rs864745						
C allele Non-Carriers	Reference	0.497		Reference	0.952	
C allele Carriers	0.51 (0.03,4.06)			0.90 (0.04,9.74)		
Female						
Dietary iron (mg/day)						
Q1 (<11.61)	Reference	0.514	0.822	Reference	0.068	0.995
Q2 (11.61-16.00)	0.47 (0.10,1.82)			0.38 (0.08,1.52)		
Q3 (16.00-19.87)	0.60 (0.15,2.14)			0.47 (0.11,1.74)		
Q4 (≥19.87)	0.16 (0.01,0.97)			0.10 (0.01,0.70)		
Dietary iron (mg/day)						
C allele Non-Carriers	Reference	0.636		Reference	0.616	
C allele Carriers	0.27 (0.01,1.58)			0.27 (0.01,1.65)		

[1] C allele presence of rs864745 was coded as 0 for non-presence and 1 for presence. Dietary iron was categorized into four groups based on quartiles, each including its lower boundary, with Q1 serving as the reference. [2] Model 1 was adjusted for age. [3] Model 2 was adjusted for age, sex, income, BMI, educational, intentional physical exercise, smoking status, alcohol use, and total dietary energy. [4] OR (95% CI) for Dietary Iron represents the risk of elevated fasting glucose occurrence of the current dietary iron range compared with the reference group. OR (95% CI) for rs864745 represented the multiplicative increase in the risk of Elevated Fasting Glucose for individuals carrying the C allele, compared to those who do not carry the C allele. [5] p_{trend}, the p value for the trend was examined using the medians in each quartile of dietary iron.[6] p_{INTM}, the p value of multiplicative interaction.

Table S5. Odds Ratios (ORs) with 95% Confidence Intervals (CIs) and Multiplicative Interaction Results for Elevated Risk of HOMA2 Index, Elevated Fasting Insulin and Fasting Glycated Hemoglobin, Based on Dietary Iron Intake and SNP rs864745, in Participants Stratified by Gender, Under Recessive Inheritance Model[1].

	Model 1[2]			Model 2[3]		
	OR (95% CI)[4]	p_{trend}[5]	p_{INTM}[6]	OR (95% CI)	p_{trend}	p_{INTM}
Elevated fasting glucose						
All						
Dietary iron						
Q1 (<12.63)	Reference	0.680	0.410	Reference	0.895	0.404
Q2 (12.63-16.53)	1.41 (0.46,4.46)			1.46 (0.47,4.68)		
Q3 (16.53-21.62)	1.09 (0.31,3.71)			1.23 (0.35,4.25)		
Q4 (≥21.62)	0.81 (0.22,2.88)			1.14 (0.29,4.21)		
rs864745						
T allele Non-Carriers	Reference	0.370		Reference	0.265	
T allele Carriers	0.67 (0.30,1.73)			0.61 (0.27,1.57)		
Male						
Dietary iron						
Q1 (<14.07)	Reference	0.619	0.431	Reference	0.963	0.453
Q2 (14.07-17.79)	0.85 (0.19,3.56)			0.89 (0.19,3.81)		
Q3 (17.79-23.59)	0.85 (0.15,3.95)			1.10 (0.19,5.34)		
Q4 (≥23.59)	0.66 (0.12,3.02)			0.98 (0.17,4.87)		
rs864745						
T allele Non-Carriers	Reference	0.159		Reference	0.104	
T allele Carriers	0.49 (0.19,1.43)			0.44 (0.17,1.28)		
Female						
Dietary iron						
Q1 (<11.61)	Reference	0.335	0.260	Reference	0.385	0.234
Q2 (11.61-16.00)	0.84 (0.14,4.60)			0.88 (0.14,5.04)		
Q3 (16.00-19.87)	0.66 (0.11,3.54)			0.72 (0.12,3.97)		
Q4 (≥19.87)	0.32 (0.02,2.63)			0.35 (0.02,2.94)		
rs864745						
T allele Non-Carriers	Reference	0.298		Reference	0.257	
T allele Carriers	0.53 (0.18,1.99)			0.50 (0.16,1.89)		
HbA1c						
All						

Dietary iron (mg/day)						
Q1 (<12.63)	Reference			Reference		
Q2 (12.63-16.53)	1.01 (0.39,2.56)	0.880		0.98 (0.37,2.58)	0.883	
Q3 (16.53-21.62)	1.07 (0.41,2.79)		0.739	1.07 (0.40,2.87)		0.838
Q4 (≥21.62)	0.90 (0.34,2.39)			1.05 (0.36,3.02)		
rs864745						
T allele Non-Carriers	Reference	0.507		Reference	0.471	
T allele Carriers	0.79 (0.39,1.58)			0.77 (0.37,1.57)		
Male						
Dietary iron (mg/day)						
Q1 (<14.07)	Reference			Reference		
Q2 (14.07-17.79)	1.61 (0.49,5.41)	0.728		1.97 (0.57,7.04)	0.570	
Q3 (17.79-23.59)	1.87 (0.51,7.09)		0.600	2.34 (0.60,9.38)		0.902
Q4 (≥23.59)	0.68 (0.18,2.46)			1.29 (0.32,5.15)		
rs864745						
T allele Non-Carriers	Reference	0.611		Reference	0.343	
T allele Carriers	1.24 (0.54,2.95)			1.54 (0.64,3.89)		
Female						
Dietary iron (mg/day)						
Q1 (<11.61)	Reference			Reference		
Q2 (11.61-16.00)	1.34 (0.30,6.25)	0.427		1.25 (0.22,6.99)	0.206	
Q3 (16.00-19.87)	0.78 (0.19,3.16)		0.309	0.63 (0.13,2.80)		0.119
Q4 (≥19.87)	0.61 (0.13,2.87)			0.39 (0.07,2.09)		
rs864745						
T allele Non-Carriers	Reference	0.234		Reference	0.127	
T allele Carriers	0.52 (0.17,1.49)			0.39 (0.10,1.23)		
HOMA-β						
All						
Dietary iron (mg/day)						
Q1 (<12.63)	Reference			Reference		
Q2 (12.63-16.53)	0.64 (0.19,2.00)	0.706		0.74 (0.22,2.42)	0.890	
Q3 (16.53-21.62)	0.74 (0.22,2.40)		0.717	0.67 (0.20,2.23)		0.677
Q4 (≥21.62)	1.18 (0.34,4.09)			1.17 (0.31,4.47)		
rs864745						
C allele Non-Carriers	Reference	0.849		Reference	0.913	
C allele Carriers	0.92 (0.35,2.16)			1.05 (0.39,2.52)		
Male						
Dietary iron (mg/day)						
Q1 (<14.07)	Reference	0.213	0.139	Reference	0.358	0.162

Q2 (14.07-17.79)	0.55 (0.14,2.10)			0.48 (0.11,1.97)		
Q3 (17.79-23.59)	0.76 (0.19,3.15)			0.64 (0.14,2.89)		
Q4 (≥23.59)	3.45 (0.67,26.42)			2.80 (0.49,22.88)		
rs864745						
T allele Non-Carriers	Reference	0.807		Reference	0.733	
T allele Carriers	1.13 (0.40,2.89)			1.20 (0.39,3.30)		
Female						
Dietary iron (mg/day)			0.294			0.440
Q1 (<11.61)	Reference	0.335		Reference	0.314	
Q2 (11.61-16.00)	3.23 (0.34,72.26)			3.28 (0.33,76.03)		
Q3 (16.00-19.87)	1.03 (0.16,6.80)			0.76 (0.11,5.19)		
Q4 (≥19.87)	0.51 (0.08,3.08)			0.50 (0.07,3.57)		
rs864745						
T allele Non-Carriers	Reference	0.992		Reference	0.995	
T allele Carriers	0.99 (0.21,3.46)			1.00 (0.21,3.63)		
HOMA2-IR						
All						
Dietary iron (mg/day)			0.699			0.805
Q1 (<12.63)	Reference	0.836		Reference	0.891	
Q2 (12.63-16.53)	1.85 (0.33,14.18)			1.85 (0.33,14.32)		
Q3 (16.53-21.62)	1.40 (0.22,11.30)			1.35 (0.20,11.09)		
Q4 (≥21.62)	0.93 (0.10,8.18)			1.34 (0.15,12.32)		
rs864745						
Non-C allele carriers	Reference	0.803		Reference	0.980	
C allele carriers	1.21 (0.35,7.62)			1.07 (0.30,6.84)		
Male						
Dietary iron (mg/day)			0.707			0.845
Q1 (<14.07)	Reference	0.814		Reference	0.843	
Q2 (14.07-17.79)	0.00 (0.00,97.42)			0.00 (0.00,260.85)		
Q3 (17.79-23.59)	0.67 (0.03,7.60)			0.85 (0.04,10.07)		
Q4 (≥23.59)	0.63 (0.03,7.17)			1.19 (0.05,14.44)		
rs864745						
T allele Non-Carriers	Reference	0.980		Reference	0.686	
T allele Carriers	0.92 (0.25,6.00)			0.73 (0.19,4.83)		
Female						
Dietary iron (mg/day)			0.789			0.639
Q1 (<11.61)	Reference	0.868		Reference	0.743	
Q2 (11.61-16.00)	3.81 (0.41,84.20)			5.11 (0.52,117.34)		
Q3 (16.00-19.87)	1.82 (0.15,42.11)			1.56 (0.13,37.10)		
Q4 (≥19.87)	1.11 (0.04,30.51)			0.98 (0.03,27.96)		
Dietary iron (mg/day)						
T allele Non-Carriers	Reference	0.893		Reference	0.893	

T allele Carriers	1.15 (0.21,21.45)			1.16 (0.21,21.68)		
Insulin						
All						
Dietary iron (mg/day)						
Q1 (<12.63)	Reference			Reference		
Q2 (12.63-16.53)	0.88 (0.03,22.88)	0.976		0.68 (0.03,18.15)	0.971	
Q3 (16.53-21.62)	0.86 (0.03,22.48)		0.990	0.65 (0.02,17.68)		0.833
Q4 (≥21.62)	0.95 (0.04,25.02)			1.04 (0.04,29.19)		
rs864745						
T allele Non-Carriers	Reference	0.512		Reference	0.410	
T allele Carriers	0.50 (0.09,9.27)			0.41 (0.07,7.76)		
Male						
Dietary iron (mg/day)						
Q1 (<14.07)	Reference			Reference		
Q2 (14.07-17.79)	0.98 (0.71,1.34)	0.884		1.12 (0.84,1.49)	0.572	
Q3 (17.79-23.59)	1.26 (0.94,1.70)		0.839	0.99 (0.71,1.37)		0.628
Q4 (≥23.59)	1.31 (0.05,34.99)			2.90 (0.09,91.09)		
rs864745	0.23 (0.03,4.71)			0.14 (0.01,3.34)		
T allele Non-Carriers	Reference	0.208		Reference	0.132	
T allele Carriers						
Female						
Dietary iron (mg/day)						
Q1 (<11.61)	Reference			Reference		
Q2 (11.61-16.00)	1.10 (0.84,1.44)	0.477		1.31 (0.89,1.91)	0.327	
Q3 (16.00-19.87)	0.72 (0.03,19.57)		0.440	0.40 (0.01,11.59)		0.409
Q4 (≥19.87)	1.19 (0.91,1.55)			1.14 (0.87,1.50)		
Dietary iron (mg/day)	0.22 (0.03,4.40)			0.19 (0.03,3.77)		
T allele Non-Carriers	Reference	0.182		Reference	0.142	
T allele Carriers						

[1] T allele presence of rs864745 was coded as 0 for non-presence and 1 for presence. Dietary iron was categorized into four groups based on quartiles, each including its lower boundary, with Q1 serving as the reference. [2] Model 1 was adjusted for age. [3] Model 2 was adjusted for age, sex, income, BMI, educational, intentional physical exercise, smoking status, alcohol use, and total dietary energy. [4] OR (95% CI) for Dietary Iron represents the risk of elevated fasting glucose occurrence of the current dietary iron range compared with the reference group. OR (95% CI) for rs864745 represented the multiplicative increase in the risk of Elevated Fasting Glucose for individuals carrying the C allele, compared to those who do not carry the C allele. [5] p_{trend}, the p value for the trend was examined using the medians in each quartile of dietary iron.[6] p_{INTM}, the p value of multiplicative interaction.

Table S6. Odds Ratios (ORs) with 95% Confidence Intervals (CIs) and Multiplicative Interaction Results for Elevated Risk of HOMA2 Index, Elevated Fasting Insulin and Fasting Glycated Hemoglobin, Based on Dietary Iron Intake and SNP rs864745, in Participants Stratified by Gender, Under a Codominant Inheritance Model[1].

	Model 1[2]			Model 2[3]		
	**OR (95% CI)[4]**	p_{trend}[5]	p_{INTM}[6]	**OR (95% CI)**	p_{trend}	p_{INTM}
Elevated fasting glucose						
All						
Dietary iron (mg/day)						
Q1 (<12.63)	Reference			Reference		
Q2 (12.63-16.53)	0.94 (0.64,1.36)	0.091		1.03 (0.69,1.53)	0.003	
Q3 (16.53-21.62)	1.08 (0.75,1.55)			1.30 (0.87,1.95)		
Q4 (≥21.62)	1.27 (0.89,1.82)		0.466	1.85 (1.19,2.89)		0.359
rs864745	0.98 (0.65,1.46)					
TT	Reference			Reference		
TC	1.16 (0.45,2.67)	0.597		1.18 (0.79,1.79)	0.453	
CC	1.27 (0.74,2.17)			1.27 (0.74,2.17)		
Male						
Dietary iron (mg/day)						
Q1 (<14.07)	Reference			Reference		
Q2 (14.07-17.79)	1.36 (0.80,2.32)	0.050		1.61 (0.92,2.86)	0.003	
Q3 (17.79-23.59)	1.49 (0.90,2.51)			1.94 (1.08,3.52)		
Q4 (≥23.59)	1.67 (1.02,2.78)		0.051	2.66 (1.42,5.05)		0.034
rs864745						
TT	Reference			Reference		
TC	1.30 (0.68,2.45)	0.045		1.33 (0.66,2.60)	0.029	
CC	2.06 (0.68,5.53)			2.40 (0.79,6.57)		
Female						
Dietary iron (mg/day)						
Q1 (<11.61)	0.78 (0.46,1.31)		0.666	0.79 (0.45,1.36)		0.747
Q2 (11.61-16.00)	0.81 (0.49,1.35)	0.808		0.85 (0.49,1.47)	0.491	
Q3 (16.00-19.87)	0.96 (0.58,1.59)			1.12 (0.61,2.09)		
Q4 (≥19.87)						

rs864745						
TT	Reference			Reference		
TC	0.88 (0.53,1.47)	0.431		0.41 (0.19,0.84)	0.532	
CC	0.41 (0.19,0.84)			1.44 (0.38,4.54)		
HbA1c						
All						
Dietary iron (mg/day)						
Q1 (<12.63)	Reference			Reference		
Q2 (12.63-16.53)	1.10 (0.84,1.44)	0.277		1.02 (0.69,1.51)	0.126	
Q3 (16.53-21.62)	1.19 (0.91,1.55)			1.29 (0.88,1.90)		
Q4 (≥21.62)	1.15 (0.87,1.50)		0.418	1.37 (0.94,2.01)		0.369
rs864745						
TT	Reference			Reference		
TC	0.94 (0.67,1.31)	0.533		1.28 (0.78,2.08)	0.478	
CC	1.24 (0.62,2.51)			0.87 (0.36,2.04)		
Male						
Dietary iron (mg/day)						
Q1 (<14.07)	1.02 (0.69,1.51)			1.05 (0.69,1.60)		
Q2 (14.07-17.79)	1.29 (0.88,1.90)	0.087		1.40 (0.91,2.16)	0.037	
Q3 (17.79-23.59)	1.37 (0.94,2.01)		0.285	1.64 (1.02,2.65)		0.229
Q4 (≥23.59)						
rs864745						
Non-C allele carriers	Reference			Reference		
	1.28 (0.78,2.08)	0.105		1.29 (0.77,2.17)	0.038	
C allele carriers	0.87 (0.36,2.04)			0.70 (0.27,1.72)		
Female						
Dietary iron (mg/day)						
Q1 (<11.61)	Reference			Reference		
Q2 (11.61-16.00)	1.30 (0.89,1.91)	0.103		1.31 (0.88,1.96)	0.285	
Q3 (16.00-19.87)	1.07 (0.74,1.56)		0.919	1.10 (0.73,1.65)		0.846
Q4 (≥19.87)	1.16 (0.79,1.69)			1.25 (0.79,1.98)		
rs864745						
TT						
TC	0.92 (0.58,1.44)	0.494		0.91 (0.57,1.44)	0.791	
CC	1.86 (0.64,5.80)			2.49 (0.77,9.61)		
HOMA-β						

All						
Dietary iron (mg/day)						
Q1 (<12.63)						
Q2 (12.63-16.53)	1.08 (0.77,1.51)	0.667		1.06 (0.74,1.51)	0.578	
Q3 (16.53-21.62)	1.17 (0.84,1.64)		0.542	1.07 (0.74,1.54)		0.590
Q4 (≥21.62)	1.09 (0.79,1.51)			0.92 (0.62,1.37)		
rs864745						
TT	Reference			Reference		
TC	1.32 (0.87,2.04)	0.722		1.36 (0.88,2.14)	0.803	
CC	1.19 (0.50,3.17)			1.05 (0.43,2.81)		
Male						
Dietary iron (mg/day)						
Q1 (<14.07)						
Q2 (14.07-17.79)	0.98 (0.62,1.56)	0.161		0.94 (0.57,1.55)	0.064	
Q3 (17.79-23.59)	0.80 (0.51,1.24)		0.194	0.70 (0.42,1.16)		0.198
Q4 (≥23.59)	0.81 (0.52,1.25)			0.68 (0.39,1.17)		
rs864745						
TT						
TC	0.81 (0.47,1.41)	0.154		0.77 (0.43,1.40)	0.124	
CC	0.82 (0.31,2.34)			0.76 (0.27,2.36)		
Female						
Dietary iron (mg/day)						
Q1 (<11.61)						
Q2 (11.61-16.00)	1.47 (0.91,2.41)	0.184		1.39 (0.83,2.34)	0.936	
Q3 (16.00-19.87)	1.27 (0.80,2.04)		0.075	1.15 (0.69,1.92)		0.112
Q4 (≥19.87)	1.46 (0.90,2.40)			1.04 (0.57,1.91)		
rs864745						
TT	Reference			Reference		
TC	1.56 (0.87,2.93)	0.123		1.60 (0.87,3.05)	0.171	
CC	1.16 (0.33,5.43)			1.16 (0.32,5.54)		
HOMA2-IR						
All						
Dietary iron (mg/day)						
Q1 (<12.63)	Reference		0.980	Reference		0.876
Q2 (12.63-16.53)	0.78 (0.46,1.31)	0.531		0.90 (0.52,1.57)	0.228	
Q3 (16.53-21.62)	1.02 (0.63,1.66)			1.23 (0.71,2.14)		

Q4 (≥21.62)	1.05 (0.65,1.71)			1.34 (0.72,2.50)		
rs864745						
Non-C allele carriers	Reference			Reference		
	0.71 (0.35,1.34)	0.813		0.81 (0.40,1.56)	0.990	
C allele carriers	0.75 (0.12,2.65)			0.87 (0.14,3.14)		
Male						
Dietary iron (mg/day)						
Q1 (<14.07)	Reference			Reference		
Q2 (14.07-17.79)	0.94 (0.44,1.99)	0.518		1.37 (0.60,3.20)	0.190	
Q3 (17.79-23.59)	1.20 (0.59,2.47)			1.96 (0.83,4.73)		
Q4 (≥23.59)	1.15 (0.57,2.36)		0.862	1.78 (0.70,4.61)		0.925
rs864745						
TT	Reference			Reference		
TC	1.15 (0.47,2.70)	0.955		1.31 (0.49,3.34)	0.968	
CC	1.14 (0.17,4.46)			1.52 (0.22,6.32)		
Female						
Dietary iron (mg/day)						
Q1 (<11.61)						
Q2 (11.61-16.00)	0.49 (0.22,1.04)	0.733		0.49 (0.22,1.08)	0.696	
Q3 (16.00-19.87)	0.93 (0.49,1.78)			0.92 (0.46,1.88)		
Q4 (≥19.87)	0.93 (0.48,1.79)		0.959	0.91 (0.40,2.09)		0.911
Dietary iron (mg/day)						
Non-C allele carriers						
	0.51 (0.18,1.24)	0.920		0.56 (0.20,1.40)	0.940	
C allele carriers	0.72 (0.04,3.93)			0.73 (0.04,4.13)		
Insulin						
All						
Dietary iron (mg/day)						
Q1 (<12.63)	Reference			Reference		
Q2 (12.63-16.53)	0.18 (0.03,0.69)	0.330		0.19 (0.03,0.74)	0.259	
Q3 (16.53-21.62)	0.84 (0.33,2.07)		0.150	0.79 (0.29,2.14)		0.157
Q4 (≥21.62)	0.37 (0.10,1.10)			0.29 (0.07,1.09)		
rs864745						
TT		0.271			0.290	

TC	0.00 (0.00,228898027.32)			0.00 (0.00,34543805.23)		
CC	1.32 (0.07,7.19)			1.61 (0.09,9.19)		
Male						
Dietary iron (mg/day)						
Q1 (<14.07)						
Q2 (14.07-17.79)	0.93 (0.17,5.08)	0.840		1.76 (0.27,14.16)	0.854	
Q3 (17.79-23.59)	0.93 (0.17,5.07)			1.75 (0.24,15.64)		
Q4 (≥23.59)	0.85 (0.16,4.64)		0.903	1.30 (0.15,13.30)		0.959
rs864745						
TT	Reference			Reference		
TC	1.11 (0.74,1.66)	0.890		1.07 (0.74,1.56)	0.953	
CC	2.87 (0.14,23.65)			4.80 (0.21,56.92)		
Female						
Dietary iron (mg/day)						
Q1 (<11.61)	Reference			Reference		
Q2 (11.61-16.00)	0.47 (0.10,1.81)	0.247		0.39 (0.08,1.53)	0.103	
Q3 (16.00-19.87)	0.60 (0.15,2.14)			0.47 (0.11,1.74)		
Q4 (≥19.87)	0.16 (0.01,0.97)		0.055	0.10 (0.01,0.70)		0.050
rs864745						
TT	Reference			Reference		
TC	1.26 (0.80,2.00)	0.145		1.32 (0.88,1.96)	0.126	
CC	2.85 (0.14,18.70)			3.29 (0.16,22.79)		

[1] rs864745 is included as an unordered multicategory variable, with categories TT, TC, and CC [2] Model 1 was adjusted for age. [3] Model 2 was adjusted for age, sex, income, BMI, educational, intentional physical exercise, smoking status, alcohol use, and total dietary energy. [4] OR (95% CI) for Dietary Iron represents the risk of elevated fasting glucose occurrence of the current dietary iron range compared with the reference group. OR (95% CI) for rs864745 represented the multiplicative increase in the risk of Elevated Fasting Glucose for individuals carrying the C allele, compared to those who do not carry the C allele. [5] p_{trend}, the *p* value for the trend was examined using the medians in each quartile of dietary iron.[6] p_{INTM}, the *p* value of multiplicative interaction.

Table S7. Odds ratios (ORs) (95% CI) and multiplicative interaction results for risk of elevated fasting glucose according to dietary iron and the SNP rs864745 in the participants stratified by sex[1]

	Model 1			Model 2		
	**OR (95% CI)[4]**	p_{trend}[5]	p_{INTM}[6]	**OR (95% CI)**	p_{trend}	p_{INTM}
All						
Dietary iron	1.00 (0.99,1.01)	0.519	0.719	1.01 (1.00,1.02)	0.022	0.786
rs864745	1.04 (0.71,1.57)	0.849		1.05 (0.70,1.60)	0.819	
Male						
Dietary iron	1.00 (0.99,1.01)	0.653	0.274	1.01 (1.00,1.02)	0.015	0.027
rs864745	1.51 (0.85,2.93)	0.189		1.71 (1.43,2.48)	0.042	
Female						
Dietary iron	1.01 (0.99,1.03)	0.391	0.779	1.02 (1.00,1.04)	0.053	0.516
rs864745	0.77 (0.42,1.44)	0.405		0.73 (0.39,1.36)	0.314	

[1] C allele presence of rs864745 was coded as 1 for presence and 0 for non-presence. [2] Model 1 was adjusted for age. [3] Model 2 was adjusted for age, sex, income, BMI, educational, intentional physical exercise, smoking status, alcohol use, and total dietary energy. [4] OR for Dietary Iron represented the multiplicative increase in the risk of Elevated Fasting Glucose for every 1 mg/day increase in Dietary Iron. OR for rs864745 represented the multiplicative increase in the risk of Elevated Fasting Glucose for individuals carrying the C allele, compared to those who do not carry the C allele. [5] p_{trend}, the p value for the trend was examined using the medians in each quartile of dietary iron.[6] p_{INTM}, the p value of multiplicative interaction.
